# Trends in fertility preservation treatments in Japan until 2023: analysis of the Japan Oncofertility Registry

**DOI:** 10.1007/s10147-025-02725-1

**Published:** 2025-02-28

**Authors:** Takao Kawai, Miyuki Harada, Yoko Urata, Yuko Sanada, Youtaro Kaneda, Yasushi Takai, Yutaka Osuga, Nao Suzuki

**Affiliations:** 1 Department of Obstetrics and Gynecology, Faculty of Medicine, The University of Tokyo, 7-3-1, Hongo, Bunkyo, Tokyo, 113-8655 Japan; 2 Division of Reproductive Medicine, Department of Perinatal and Maternal Care, National Center for Child Health and Development, Tokyo, Japan; 3 Department of Obstetrics and Gynecology, Saitama Medical Center, Saitama Medical University, Kawagoe, Japan; 4 Department of Obstetrics and Gynecology, St. Marianna University School of Medicine, Kawasaki, Japan

**Keywords:** Fertility preservation, Japan Oncofertility Registry, Cancer statistics

## Abstract

**Background:**

Fertility preservation for patients with cancer or other diseases who receive gonadotoxic treatment has gained importance as cancer survival rates increase. In Japan, a database for registering all fertility preservation patients, named the Japan Oncofertility Registry (JOFR), was established in 2018. This study aimed to analyze recent trends in fertility preservation in Japan utilizing data from the JOFR.

**Methods:**

Data was extracted from the JOFR for patients who consulted fertility preservation teams until May 2024. A descriptive analysis was conducted to examine trends in patient demographics, cancer types, fertility preservation treatments, complications, and outcomes. The data covered the period from diagnosis to fertility preservation and subsequent usage or disposal of frozen specimens.

**Results:**

A total of 11,510 patients were recorded, with 9491 undergoing fertility preservation treatments. The number of patients increased steadily after 2006. After 2021, the number of female patients was much higher than the number of male patients. The most common primary diseases were breast cancer among women and testicular tumors and leukemia among men. There were some complications including ovarian hyperstimulation syndrome (5.0%), bleeding (0.12%), and infections (0.05%) for women. Seven hundred and sixty clinical pregnancies were recorded, with 440 using preserved specimens. The discard rate was 16.3% for men and 3.7% for women.

**Conclusion:**

The study highlights recent trends in the growing number of cases undergoing fertility preservation in Japan. It also identifies several issues to be solved in fertility preservation in Japan, regarding its efficacy and safety, as well as the medical provision system.

**Supplementary Information:**

The online version contains supplementary material available at 10.1007/s10147-025-02725-1.

## Introduction

Recently, remarkable advances in cancer treatment have led to increased cancer survival rates [1]. Consequently, there is a growing need to consider the post-treatment lives of cancer survivors. Some anticancer drugs and radiation therapies have long-term complications, including effects on growth and development, secondary cancers, and infertility, such as premature menopause and azoospermia [2]. While cell damage caused by anticancer drugs and radiation is reversible in tissues with a high regenerative potential, such as bone marrow and the gastrointestinal mucosa, damage to ovaries is permanent. In light of these trends, fertility preservation has become increasingly important to improve the reproductive outcomes of cancer survivors. Additionally, anticancer drugs and radiation therapies are sometimes used to treat certain autoimmune diseases and other benign diseases; therefore, fertility preservation for patients with these conditions should also be considered [3].

To preserve fertility, several treatments are utilized, including freezing of oocytes, sperm, embryos, and gonadal tissue before anticancer drug treatment and radiation therapies [4, 5]. Gonadotropin-releasing hormone agonists (GnRHa) are also concurrently administered with anticancer drugs to protect the ovaries. Oocyte freezing is an option for women without a partner, while embryo freezing is used for women with a partner. For prepubertal girls, oocyte retrieval is challenging and ovarian tissue cryopreservation is employed. GnRHa are used to protect the ovaries when time is insufficient for other methods, although evidence regarding their effectiveness for fertility preservation remains limited [6–8]. Sperm freezing is standard for men, but testicular tissue cryopreservation is performed for prepubertal boys who do not yet ejaculate [9, 10]. In total, 6–24% and 50% of testicular cancer patients are reported to be azoospermic and oligozoospermic at the time of diagnosis, respectively [11]. Consequently, sperm freezing is sometimes performed not by ejaculation but by extracting sperm from testicular tissue during surgery, a procedure known as onco-testicular sperm extraction (onco-TESE).

Despite the growing demand for fertility preservation worldwide, an international online survey of reproductive physicians between 2016 and 2017 revealed that approximately 20 percent of patients sought fertility preservation advice independently, without an oncologist's referral. This highlights the need for greater collaboration between oncologists and reproductive physicians [12]. To better understand the current situation in each country, the establishment of public registries documenting the short- and long-term outcomes of fertility preservation treatments has been strongly recommended [13]. While not many countries have their own national oncofertility registry, some, such as Germany, Austria, Switzerland, Australia, and New Zealand, have established national registries [14]. In Japan, fertility preservation treatment is gaining popularity among young cancer patients [15]. In 2018, the Japan Society for Fertility Preservation established a database to register all fertility preservation patients, named the Japan Oncofertility Registry (JOFR). In 2021, fertility preservation treatments for patients younger than 43 years became eligible for public subsidies, although some treatments, such as GnRHa and testicular tissue cryopreservation, are not covered [16]. Previous research reported the national demographics of fertility preservation, including the percentage of adolescent and young adult cancer patients opting for fertility preservation, using questionnaire surveys [17]. Another study provided a pilot analysis of reproductive outcomes following fertility preservation in Japan using questionnaire surveys [18]. Recent trends in fertility preservation for patients with various types of cancer have been reported [19–21]. Furthermore, studies using data from the JOFR have provided basic information on fertility preservation, such as age, type of cancer, and pregnancy rates up to 2021 [22]. However, there has been no further comprehensive epidemiological research of fertility preservation, and the trends in fertility preservation in Japan remain unclear.

To understand these trends, we used data from the JOFR. Almost all fertility preservation patients are registered in the JOFR because registration is mandatory for patients and facilities to receive public subsidies. This study aimed to comprehensively elucidate recent trends in fertility preservation in Japan.

## Patients and methods

### Patients

Patients who consulted a fertility facility for fertility preservation were registered in the JOFR. Informed consent was obtained at individual facilities. Patients' information, including their demographic data, cancer type, fertility preservation treatments, and usage of frozen specimens, was registered in the JOFR by patients using an app and by their fertility clinic online.

### Data extraction

The following data were extracted from the JOFR up to 2023 in May 2024: age; year of fertility preservation; sex; residence; type of cancer; marital status; method of fertility preservation; number of frozen specimens; complications of fertility preservation; periods from diagnosis to cancer treatment, consultation for fertility preservation, and fertility preservation treatment; recurrence; usage of frozen specimens; disposal of frozen specimens; pregnancy rate; and mortality rate. A descriptive analysis was conducted to understand recent trends in fertility preservation using JMP Pro 17. All registered items were used in each case and missing data was omitted in each analysis.

### Ethical approval

Patient enrollment was approved by the ethics committee of each institution or collectively reviewed by the ethics committee of Tohoku University (ethics number: UMIN000045292).

## Results

A total of 11,510 patients had initial consultations with a fertility preservation team until the end of 2023. Among these, 9491 patients (4799 women and 4692 men) proceeded with fertility preservation treatment, while the remaining patients received only consultations without treatment. The key demographic data among 9491 patients was shown in Table 1.

**Table 1:** Facilities and patients’ demographic characteristics

Facilities registered (hospitals or clinics) (number)	155	
Patients’ demographic characteristics		
Gender (number)	Male 4692	Female 4799
Age (years)	32 (median) (quartile range 25–37)	
Marriage status^a^ (number)	Married 2771	Unmarried 5418
Alive of dead (at the time of data extraction) (number)^a^	Alive 8193	Dead 349
Primary cancer (or disease) or recurrent cancer (number)^a^	Primary 7127	Recurrence 611

^a^Missing data was excluded

### Trends in the number of patients

**Fig. 1:**
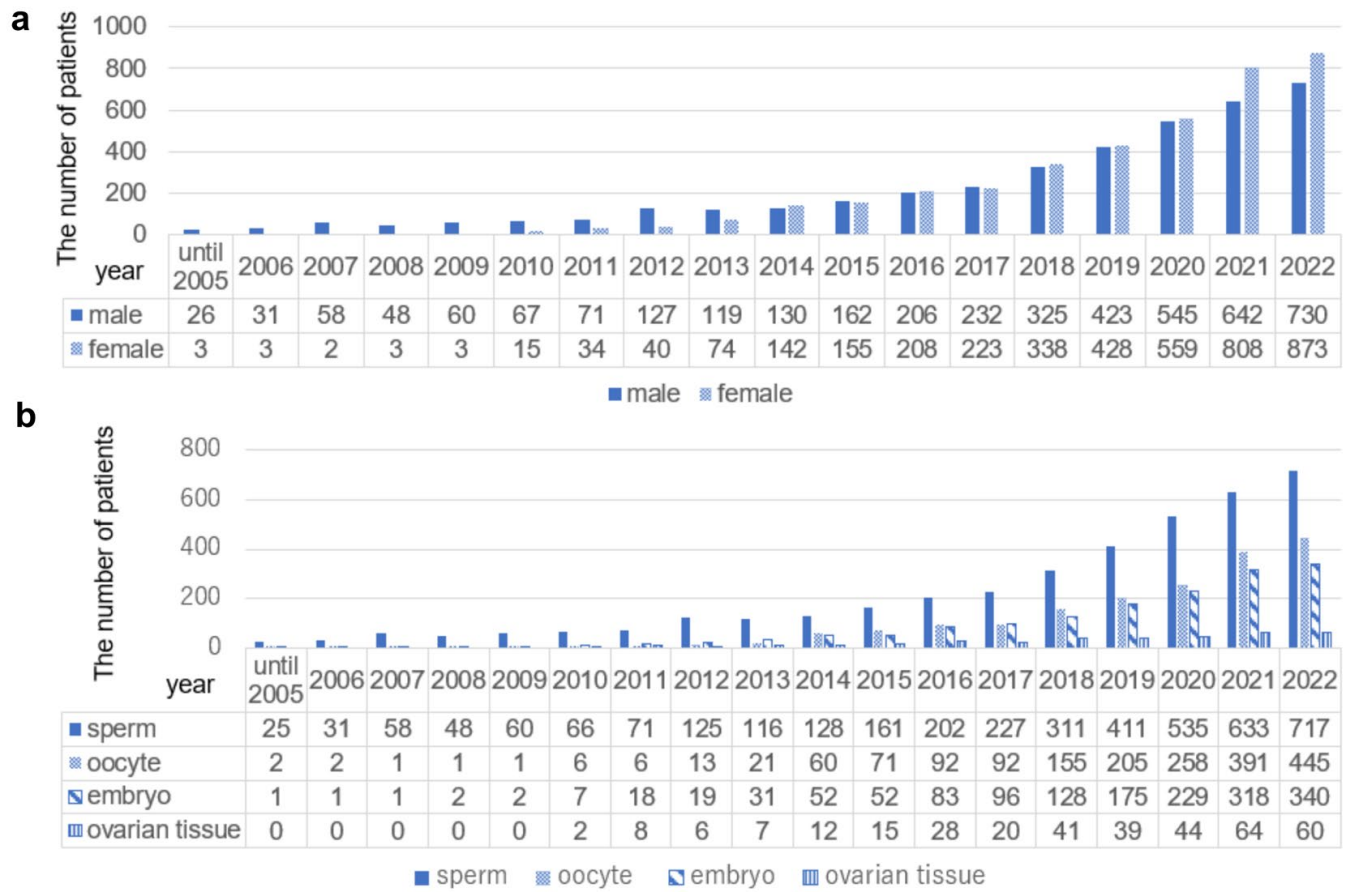
Numbers of patients undergoing fertility preservation treatment. Annual trends of fertility preservation treatment according to sex (**a**) and the method of fertility preservation treatment (**b**). Data from 2023 had not yet been completely registered in October 2024 and thus were excluded

More male patients than female patients underwent fertility preservation until 2013 (Fig. 1a). From 2014 to 2020, the number of male and female patients tends to be approximately equal. From 2021 onward, there were more female patients than male patients. Overall, the number of patients steadily increased. Not all data from 2023 had yet been registered in May 2024; therefore, patients in 2023 were excluded in Fig. 1a and b.

Sperm, oocyte, embryo, and ovarian tissue freezing were registered from 1999, 2003, 2004, and 2010, respectively (Fig. 1b). The total number of patients steadily increased, but there were several periods of rapid growth, including 2014, 2018, and 2021. The numbers of patients who underwent oocyte, embryo, and ovarian tissue freezing were 2.85 times (60/21), 1.67 times (52/31), and 1.71 times (12/7) higher in 2014 than in 2013, respectively. The year-on-year increases in the numbers of patients who underwent sperm, oocyte, embryo, and ovarian tissue freezing were 1.37 times (311/227), 1.68 times (155/92), 1.33 times (128/96), and 2.05 times (41/20) in 2018, respectively, and 1.18 times (633/535), 1.51 times (391/258), 1.38 times (318/229), and 1.45 times (64/44) in 2021, respectively.

### Numbers of patients by age group

**Fig. 2:**
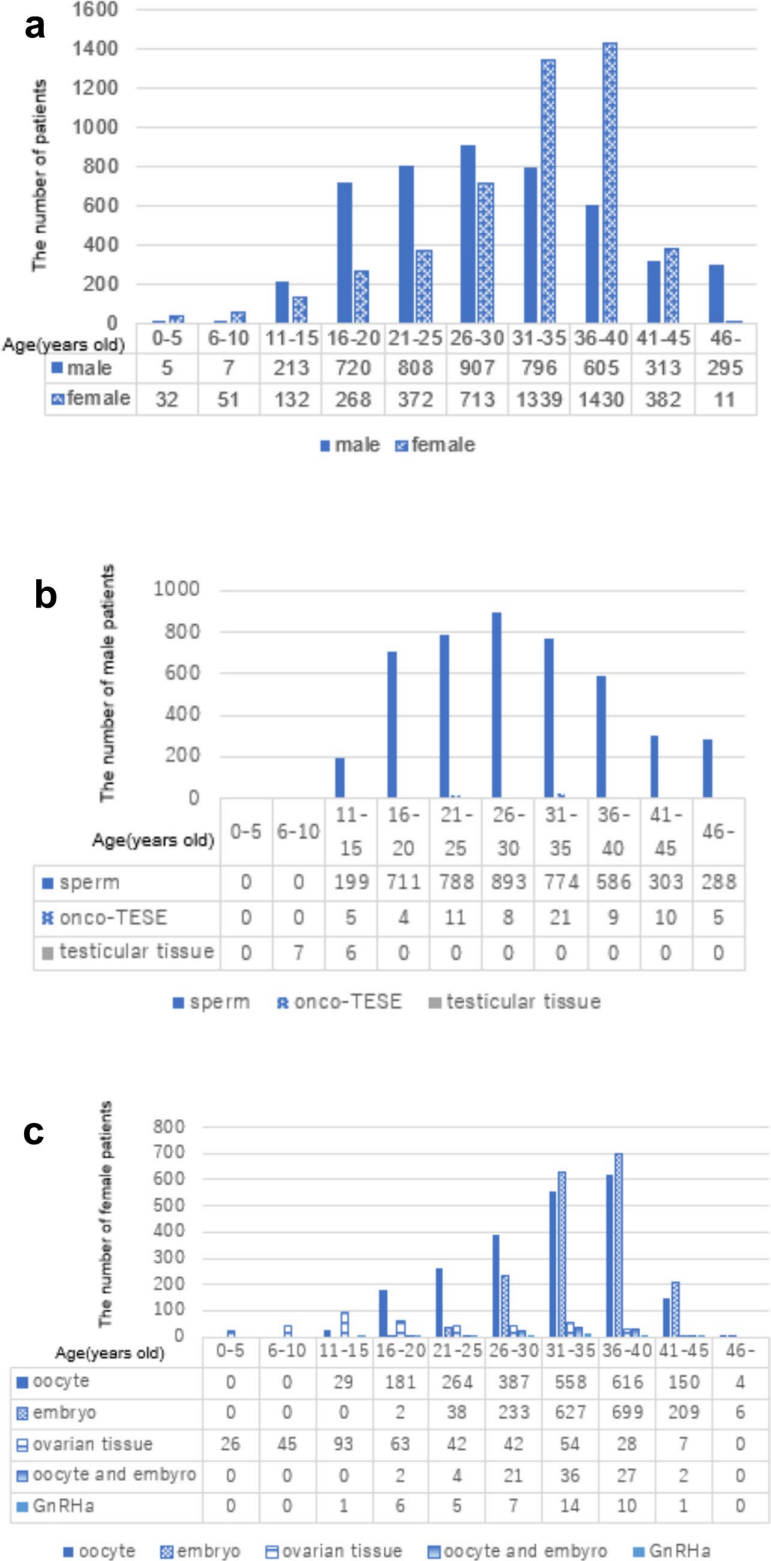
Age distributions of patients undergoing fertility preservation treatment. Age distributions of patients undergoing fertility preservation treatment according to sex (**a**) and the method of fertility preservation treatment for men (**b**) and women (**c**)

The peak age for the total number of patients was late 20s for men and late 30s for women (Fig. 2a). Among men, the peak ages for sperm freezing, onco-TESE, and testicular tissue freezing were late 20s, early 30s, and 6–10 years, respectively (Fig. 2b). Among women, the peak ages for oocyte and embryo freezing, ovarian tissue freezing, and GnRHa therapy were late 30s, early teens and late 30s, and early 30s, respectively (Fig. 2c).

### Numbers of patients undergoing fertility preservation by area

**Fig. 3:**
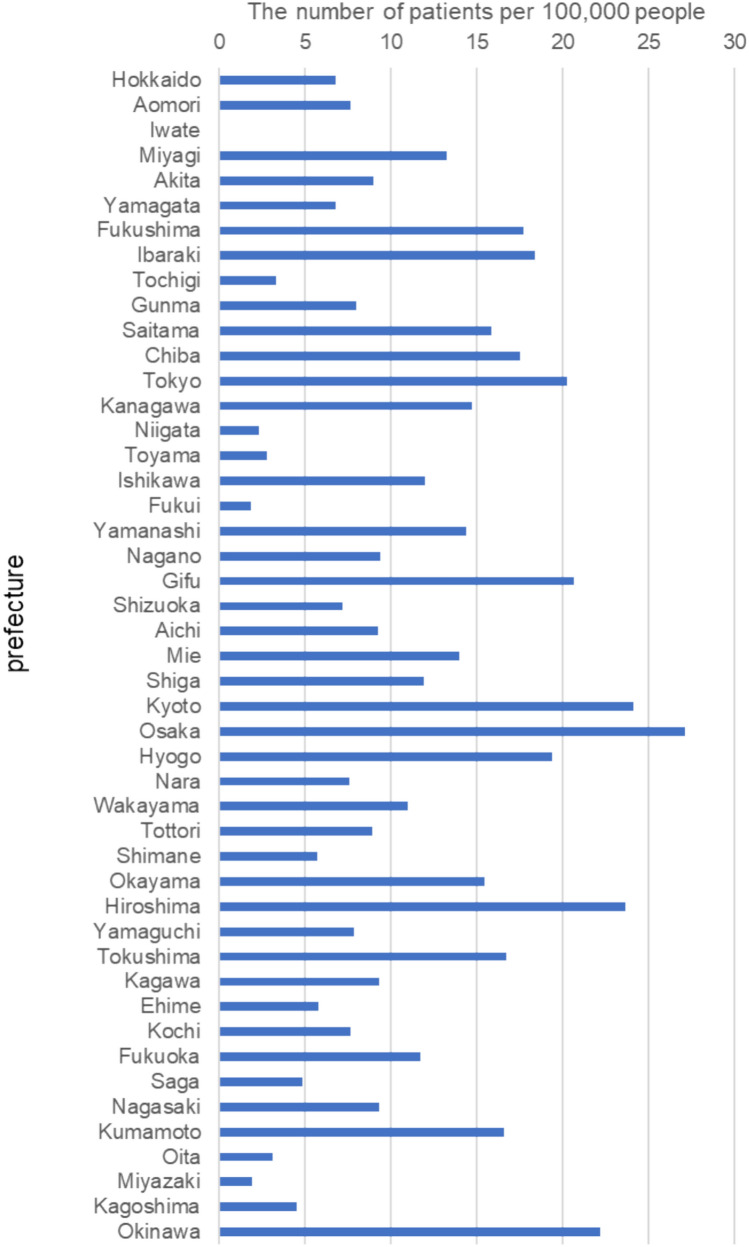
Residence of patients undergoing fertility preservation treatment. Number of patients undergoing fertility preservation treatment until 2023 per 100,000 people younger than 49 years old by prefecture

The number of patients undergoing fertility preservation until 2023 per 100,000 people younger than 49 years old by prefecture varied from 0 to 27 depending on the prefecture (Fig. 3). The prefecture population in 2022 was obtained from the national open database published by the Ministry of Internal Affairs and Communications [23].

### Primary diseases of patients undergoing fertility preservation

**Fig. 4:**
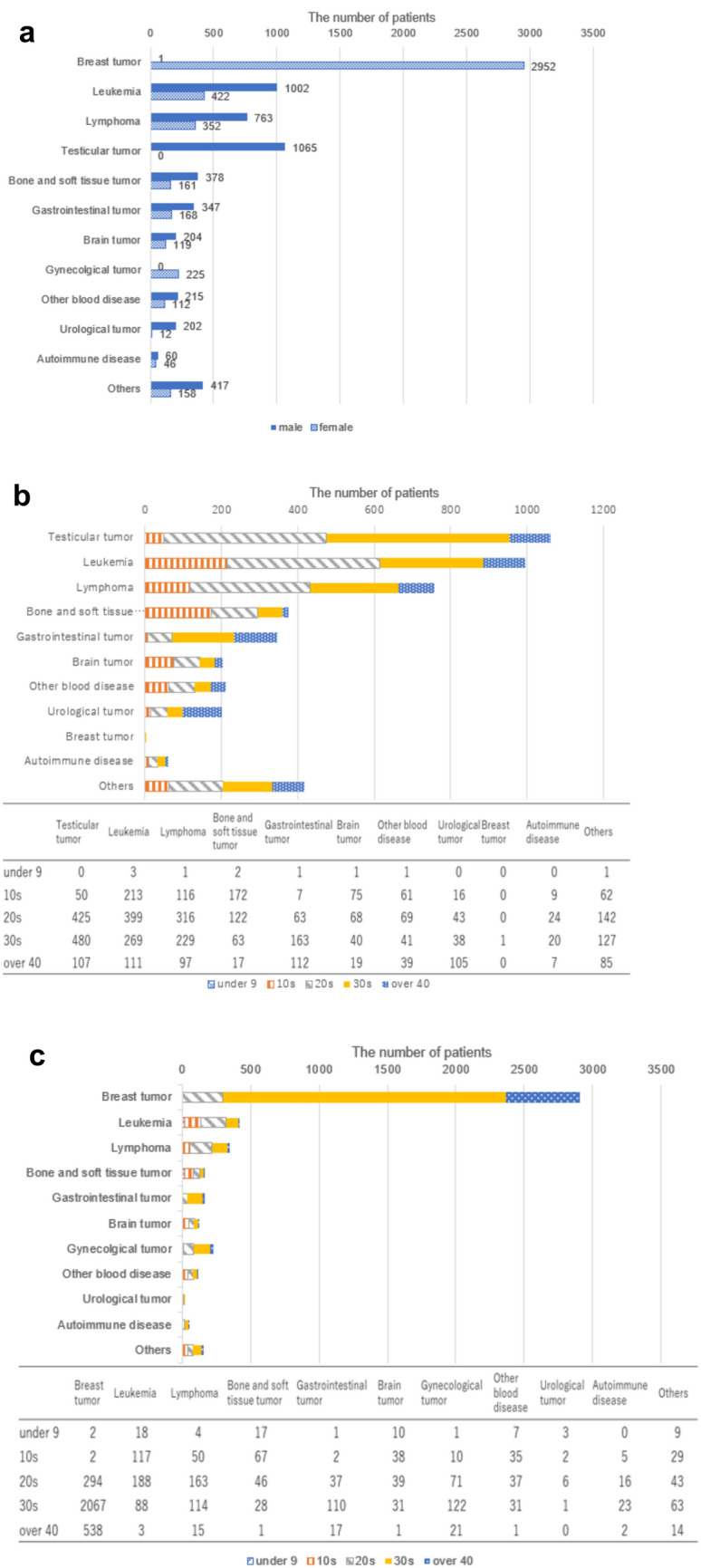
Primary diseases of patients undergoing fertility preservation treatment. Primary diseases of patients undergoing fertility preservation according to sex (**a**) and age for men (**b**) and women (**c**)

The most common primary diseases were breast cancer among women and testicular tumors and leukemia among men (Fig. 4a). The numbers of male patients with leukemia, lymphoma, bone and soft tissue tumors, gastrointestinal tumors, and urological tumors as the primary disease were more than double the numbers of female patients. Except for breast cancer, autoimmune disease, and gynecological disease, the number of male patients was more than double the number of female patients. Although there were relatively more female patients with autoimmune disease, the number of male patients was still higher (Fig. 4a). In total, 359 male patients (9.5%) and 252 female patients (6.3%) had recurrent cancer.

Among men, the most common primary disease was leukemia in those aged 9 years and younger and teens, testicular cancer in those in their 20s and 30s, and gastrointestinal tumors in those aged 40 years and older (Fig. 4b). Among women, the most common primary disease was leukemia in those aged 9 years and younger and teens, and breast cancer in those in their 20s and 30s and those aged 40 years and older (Fig. 4c).

In Fig. 4, ‘Others’ include other rare cancers affecting young adults, such as pharyngeal cancer, tongue cancer, thyroid cancer, lung cancer, skin cancer, and cancer of unknown primary origin, and other diseases that require fertility preservation treatment like chronic active Epstein–Barr virus infection, congenital immunodeficiency, Turner syndrome, and Klinefelter syndrome.

### Numbers of patients by fertility preservation treatments

For all registered patients, the most common fertility preservation treatment was sperm freezing, followed by oocyte freezing and embryo freezing (Supplementary Fig. 1).

**Fig. 5:**
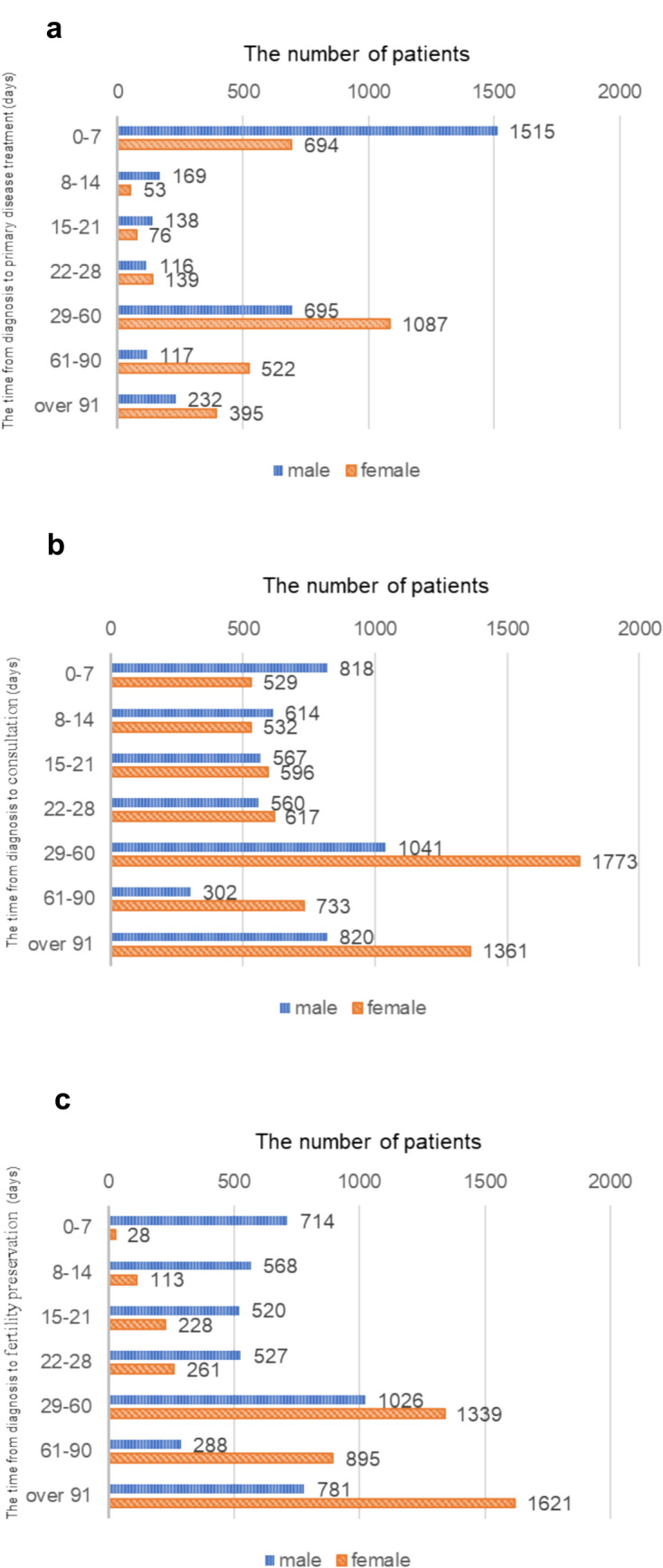
Time from diagnosis of primary disease. Times from diagnosis of primary disease to primary disease treatment (**a**), consultation with a fertility clinic (**b**), and fertility preservation treatment (**c**)

### Time from diagnosis of primary disease

The time from diagnosis of primary disease to treatment of primary disease was most commonly 0–7 days for men and 29–60 days for women (Fig. 5a). The median time was 7 days for men and 31 days for women. The time from diagnosis of primary disease to the first consultation at a fertility preservation clinic was most commonly 29–60 days for both men and women, followed by 91 days or more (Fig. 5b). The median time was 26 days for men and 40 days for women. The time from diagnosis of primary disease to completion of fertility preservation therapy was most commonly 29–60 days for men, followed by 91 days or more, and was 91 days or more for women, followed by 29–60 days (Fig. 5c). The median time was 27 days for men and 68 days for women.

**Fig. 6:**
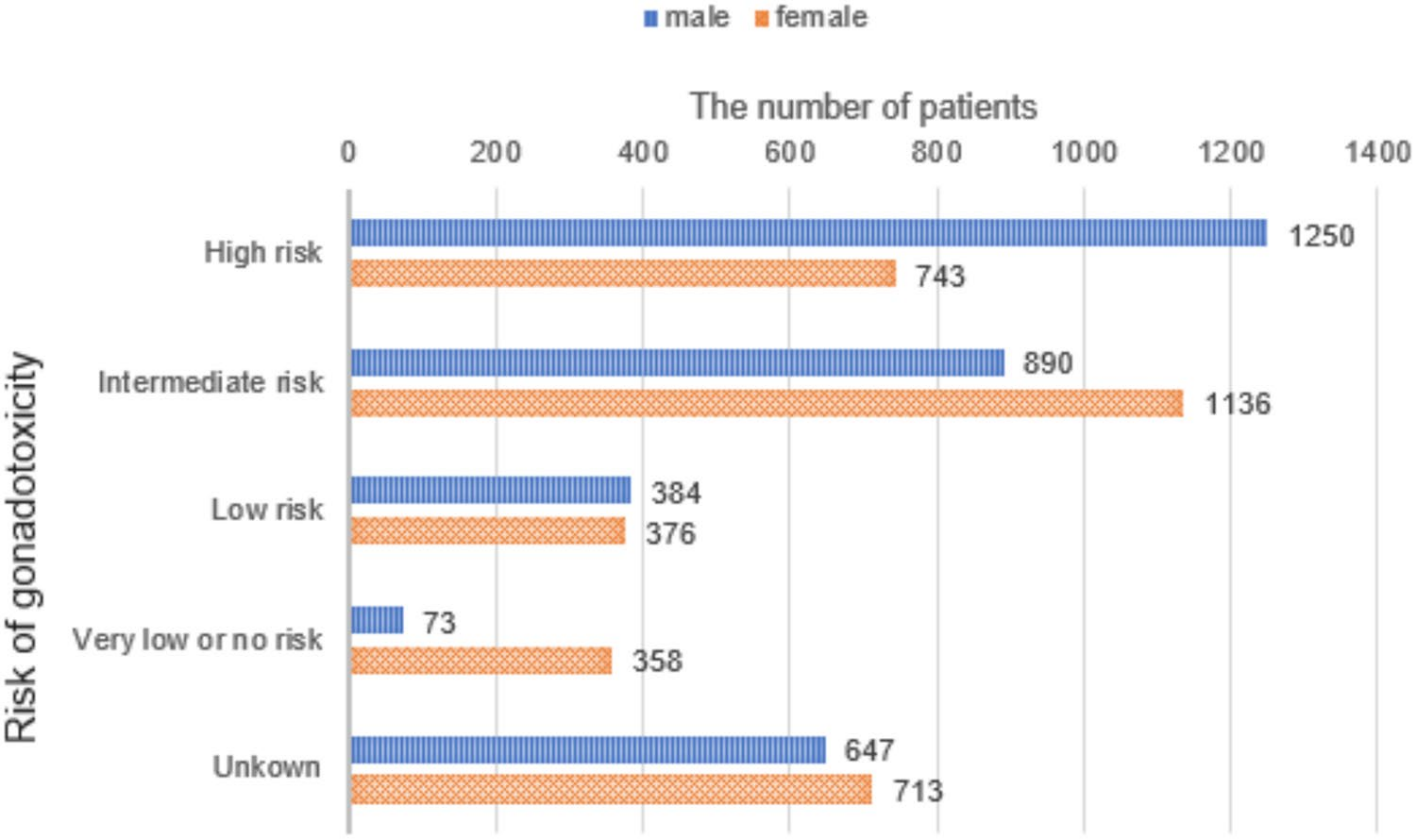
Risk of gonadotoxicity induced by primary disease treatment. Risk of gonadotoxicity induced by primary disease treatment. The risk was defined according to the American Society of Clinical Oncology’s recommendations for fertility preservation

### Gonadotoxicity caused by primary disease treatment

Based on “Effects of Different Antitumor Agents on Sperm Production in Men” and “Risks of Permanent Amenorrhea in Women Treated With Modern Chemotherapy and Radiotherapy” reported in the American Society of Clinical Oncology’s recommendations for fertility preservation [2, 24], we evaluated gonadotoxicity caused by primary disease treatment. The risk of gonadotoxicity was high, intermediate, low, very low or no, and unknown in 38.5% (1250/3244), 27.4% (890/3244), 11.8% (384/3244), 2.3% (73/3244), and 19.9% (647/3244) of men, respectively, and in 22.3% (743/3326), 34.2% (1136/3326), 11.3% (376/3326), 10.8% (358/3326), and 21.4% (713/3326) of women, respectively (Fig. 6).

### Complications of fertility preservation treatments

There were some complications of fertility preservation treatments. Among oocyte retrieval patients (n = 4163), some suffered ovarian hyperstimulation syndrome (OHSS, n = 208, 5.00%), bleeding (n = 5, 0.12%), infections (n = 2, 0.05%), and pulmonary embolism (n = 1, 0.02%). Two patients (2.7%) who underwent testicular tissue biopsy and one patient (0.02%) who underwent sperm freezing suffered from bleeding.

### Usage of specimens

The median numbers of frozen embryos and oocytes per patient were five and nine, respectively. The median number of tubes of frozen sperm per patient was six, and sperm freezing was performed a median of one time. For women, the usage rate of specimens was 10.6% (269/2536) and the discard rate was 3.7% (94/2536). For oocytes, 3.2% (42/1323) of specimens were used and 2.9% (38/1323) were discarded. For embryos, 20.9% (225/1078) of specimens were used and 4.8% (52/1078) were discarded. For ovarian tissue, 1.8% (3/166) of specimens were used and 1.8% (3/166) were discarded. For men, the usage rate of specimens was 8.6% (211/2455) and the discard rate was 16.3% (402/2455).

### Prognosis

By May 2024, clinical pregnancies were recorded for 455 female patients and 305 male patients’ partners. Among them, 264 female patients and 176 male patients’ partners underwent assisted reproductive technology (ART) treatment with frozen specimens. Two patients naturally became pregnant after frozen ovarian tissue transplants, and four patients became pregnant following ART treatment after ovarian tissue transplants. As of May 2024, 239 men and 110 women undergoing fertility preservation treatment had died. Leukemia (n = 64) and breast cancer (n = 36) were the most common causes of death for men and women, respectively.

## Discussion

In the present study, we described the comprehensive features of fertility preservation, including annual trends, age, cancer type, residence, method of fertility preservation, number of frozen specimens, period from diagnosis to consultation or fertility preservation treatment, complications, usage and discard rates, pregnancy, and death.

The annual trend showed an increase in the number of fertility preservation treatments, indicating fertility preservation is being increasingly performed. In 2012, the Japanese Society of Fertility Preservation was established. The Japan Society of Clinical Oncology Clinical Practice Guidelines 2017 for fertility preservation in childhood, adolescent, and young adult cancer patients were published in 2017 and translated into English in 2022 [25, 26]. These awareness-raising activities increased the number of patients. From 2021, far more female patients than male patients underwent fertility preservation treatments. This is possibly because public subsidies for fertility preservation started in 2021 and the financial burden of fertility preservation for women, which is usually heavier than that for men, was reduced. However, the annual number must be interpreted with caution. Registration with the JOFR became mandatory for patients applying for public subsidies in April 2021, when the public subsidies system began; therefore, data registration before 2021 may be insufficient. In addition, the JOFR was established in November 2018; therefore, data registration before 2018 was retrospective, leading to missing data. Indeed, the number of patients undergoing fertility preservation registered in the JOFR before 2019 is lower than the number identified in 2011–2015 and 2016–2019 by questionnaire surveys [27, 28]. The epidemiological trend, such as the number of annual cases of malignant disease, was not taken into account in this analysis.

Women and men most commonly underwent fertility preservation in their late 30s and late 20s, respectively. This discrepancy is due to the different types of cancer affecting men and women. Specifically, breast cancer and testicular tumors were the most common reasons for fertility preservation in women and men, respectively. The average age of breast cancer patients is older than that of testicular tumor patients [29, 30]. In terms of fertility preservation treatments, most men opted for sperm freezing, while young women (aged 10–20 years) preferred oocyte freezing and older women (aged 30–40 years) chose embryo freezing, likely based on whether they had a marital partner. Some very young girls (aged 0–10 years) underwent ovarian tissue freezing.

Regarding the prevalence of fertility preservation in different prefectures, Kyoto, Osaka, and Hiroshima had the highest numbers of patients undergoing fertility preservation relative to the population. There was a disparity in fertility preservation among prefectures. Prefectures with high fertility preservation rates are all expected to have a core hospital for fertility preservation treatment and good coordination between the core hospital and other hospitals. In the five prefectures (Iwate, Miyazaki, Fukui, Toyama, and Niigata) with the lowest numbers of patients undergoing fertility preservation, the development of cancer and reproductive medicine networks was slow. In those prefectures, the lack of transportation accessibility in the intra-region and the convenience of transportation to metropolitan in nearby prefectures might be among the reasons for the lower number of patients. However, the actual situation was not clear only from the JOFR data.

The median time from diagnosis of primary disease to consultation with a fertility preservation clinic was long. Previous research in other countries reported a shorter median time from diagnosis of primary disease to consultation (5–22 days), although they were single-center retrospective analyses [31, 32]. Variation in the time from diagnosis to consultation implied accessibility to fertility preservation treatment differed between facilities. In addition, comparison of the time from diagnosis of primary disease to the first consultation at a fertility preservation clinic with the time from diagnosis of primary disease to treatment of primary disease showed that fertility preservation was often performed after cancer treatment had started.

The most common complication of fertility preservation was OHSS, which was diagnosed with 5.0% of patients who underwent oocyte retrieval. This incidence is much higher than that among patients registered in the Japanese ART registry (less than 1.0%) [33], which is partly because the JOFR includes OHSS at all stages, while the Japanese ART registry only includes moderate or severe OHSS. It is important to avoid delays in cancer treatment due to OHSS, and fertility preservation patients may have a higher risk of OHSS because of their younger age than infertility patients. Moreover, one patient with pulmonary embolism as complication for fertility preservation treatment was reported in the JOFR. The thrombosis risk is usually higher in cancer patients, but is not increased by ovarian stimulation for fertility preservation alone [34]. Reproductive medicine physicians need to be aware of complications unique to cancer patients.

The usage rate of ovarian tissue (1.8%) is still lower in Japan than 6–30% in other countries because ovarian tissue transplantation is not yet widely and long practiced [35]. However, the number of ovarian tissue cryopreservation has been increasing during the past decade as shown in Fig. 1b and moreover, the patients undergoing ovarian tissue cryopreservation were younger than those with oocyte or embryo cryopreservation as shown in Fig. 2c, with a dominant age group of 11–15 years old, the usage rate of ovarian tissue will be higher in near future. Higher discard rate of men in comparison with women was possibly because men can undergo sperm freezing more easily than women can undergo oocyte or embryo cryopreservation. Among 760 pregnancy patients or patients’ partners, 314 patients did not use their preserved specimens, indicating that about 40% of patients may not need to preserve oocytes, embryos, or sperm to have a baby after cancer treatment. However, it is difficult to accurately assess fertility loss before cancer treatment. Accurate evaluation of patients requiring fertility preservation is a future challenge.

Compared to other countries, a unique feature of the Japanese oncofertility landscape, compared to other countries, is the establishment of oncofertility networks in each prefecture. This development is partly because public subsidies are a prerequisite for the establishment of these prefectural networks. In the Netherlands, where the population size is comparable to that of a Japanese prefecture such as Chiba, there is only a single national oncofertility network. A small number of hospitals provide cancer treatment, leading to the concentration of medical resources in a few facilities. As a result, data registries in the Netherlands maintain high-quality records with minimal missing data; however, the overall quantity of data is relatively low [36]. In Germany, where the population is two-thirds that of Japan, a centralized network called FertiPROTEKT was established. This network includes over 100 medical facilities across Germany, Switzerland, and Austria [36]. Although each facility operates independently, they are part of a centralized system. The advantages of a centralized network include an effective supervision and education system; however, a drawback is the potential delay between oncologist consultations and fertility preservation treatments. In contrast, the decentralized Japanese network offers advantages such as quicker consultations and reduced geographical burden for patients in prefectures with well-established networks. However, as shown in Fig. 3, a significant drawback of the decentralized system is the inequality in oncofertility promotion across different regions, which remains a challenge for future improvements. The JOFR registry has collected nearly all fertility preservation treatment data in Japan, making it easier to utilize national data for policy advocacy and healthcare planning.

There are several limitations of this study. The data included in the analysis were those registered in the JOFR without personal identification; thus, we did not conduct further research of each case. For example, we could not determine specific cancer treatments. Regarding patients who underwent oocyte retrieval, we did not determine the protocol applied for controlled ovarian stimulation or the preventive measure for OHSS. We also could not determine specific pregnancy outcomes like delivery method or perinatal complications. Additionally, not all data is registered in the JOFR. There may be cancer survivors who have babies but are not registered in the JOFR because patients who discard specimens and are lost to follow-up cannot be detected using JOFR data. Moreover, not all fertility preservation patients were registered because patients who were ineligible for public subsidies did not have to be registered; patients older than 43 years, those who took GnRHa or underwent testicular tissue cryopreservation as fertility preservation treatment, or those who underwent fertility preservation treatments before the public subsidies started in 2021 may not have been registered. Accordingly, there was a selection bias, as facilities with active registration tend to be more engaged in oncofertility treatment. In the future, we will supplement this potentially missing data with other research, such as questionnaire surveys, to gain a more accurate understanding. It is definitely needed to improve the quality of registries by integrating JOFR with other datasets including ART registries or national cancer statistics and by reducing missing data.

In conclusion, analysis of JOFR data clarified the recent trends in fertility preservation in Japan, although several issues must be addressed regarding this registration system. The results of the present study identified two issues with fertility preservation in Japan. The first is the efficacy and safety of fertility preservation currently performed in Japan. The indication for fertility preservation should be discussed based both on the reproductive outcome and prognosis of the primary disease. In addition, a cost-beneficial point of view is also required, given that public subsidies are applied for fertility preservation. The other is the medical provision system in Japan, including accessibility to fertility preservation services. Fertility preservation is a part of cancer treatment, not an extra benefit for specific patients; thus, it should be available to whoever needs it in a timely manner. The JOFR will greatly contribute to progress in fertility preservation in Japan. Moreover, this large-scale registry specific to fertility preservation will also contribute to progress in this relatively new area of medicine.

## Supplementary Information

Below is the link to the electronic supplementary material.

Supplementary file1 (DOCX 28 kb)

## Data Availability

The data supporting the findings of this study are available within the paper or upon reasonable request to the corresponding author.
