# Supplementary material for: Trends in fertility preservation treatments in Japan until 2023: analysis of the Japan Oncofertility Registry
Source: Int J Clin Oncol. 2025 Feb 28;30(4):684–95. doi: 10.1007/s10147-025-02725-1 (PMC11947001; doi:10.1007/s10147-025-02725-1)

**Supplementary Fig.1.** Numbers of patients undergoing fertility preservation treatment according to the method.


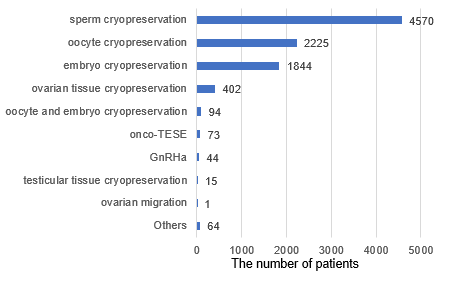

Supplement: Supplementary file 1 — Supplementary file1 (DOCX 28 kb) [file 10147_2025_2725_MOESM1_ESM.docx]
